# Refinement of Draft Genome Assemblies of Pigeonpea (*Cajanus cajan*)

**DOI:** 10.3389/fgene.2020.607432

**Published:** 2020-12-15

**Authors:** Soma S. Marla, Pallavi Mishra, Ranjeet Maurya, Mohar Singh, Dhammaprakash Pandhari Wankhede, Anil Kumar, Mahesh C. Yadav, N. Subbarao, Sanjeev K. Singh, Rajesh Kumar

**Affiliations:** ^1^Indian Council for Agricultural Research (ICAR)-National Bureau of Plant Genetic Resources, New Delhi, India; ^2^Directorate of Education, Rani Lakshmi Bai Central Agricultural University, Jhansi, India; ^3^School of Computational and Integrative Sciences, Jawaharlal Nehru University, New Delhi, India

**Keywords:** assembly improvement, reconciliation, mate-pairs, disease resistance, pigeonpea genome

## Abstract

Genome assembly of short reads from large plant genomes remains a challenge in computational biology despite major developments in next generation sequencing. Of late several draft assemblies have been reported in sequenced plant genomes. The reported draft genome assemblies of *Cajanus cajan* have different levels of genome completeness, a large number of repeats, gaps, and segmental duplications. Draft assemblies with portions of genome missing are shorter than the referenced original genome. These assemblies come with low map accuracy affecting further functional annotation and the prediction of gene components as desired by crop researchers. Genome coverage, i.e., the number of sequenced raw reads mapped onto a certain location of the genome is an important quality indicator of completeness and assembly quality in draft assemblies. The present work aimed to improve the coverage in reported *de novo* sequenced draft genomes (GCA_000340665.1 and GCA_000230855.2) of pigeonpea, a legume widely cultivated in India. The two recently sequenced assemblies, A1 and A2 comprised 72% and 75% of the estimated coverage of the genome, respectively. We employed an assembly reconciliation approach to compare the draft assemblies and merge them, filling the gaps by employing an algorithm size sorting mate-pair library to generate a high quality and near complete assembly with enhanced contiguity. The majority of gaps present within scaffolds were filled with right-sized mate-pair reads. The improved assembly reduced the number of gaps than those reported in draft assemblies resulting in an improved genome coverage of 82.4%. Map accuracy of the improved assembly was evaluated using various quality metrics and for the presence of specific trait-related functional genes. Employed pair-end and mate-pair local libraries helped us to reduce gaps, repeats, and other sequence errors resulting in lengthier scaffolds compared to the two draft assemblies. We reported the prediction of putative host resistance genes against *Fusarium* wilt disease by their performance and evaluated them both in wet laboratory and field phenotypic conditions.

## Introduction

Recent rapid developments in genome sequencing technologies have facilitated the generation of several draft assemblies in plants. These are valuable resources for elucidating genetic information and understanding the biology of the crop. However, each of these draft assemblies have strengths and weaknesses as they were sequenced and assembled based on different technologies and algorithms (Singh et al., 2012; Varshney et al., 2012). Draft assemblies differ depending on the sequencing technology and the assembly software employed. One assembly may be conservative in its selection of reads resulting in low genome coverage with many gaps. Another assembler may be vigorous, yielding more contigs but with many errors. Draft genomes are typically sets of a large contingent of assembled contigs and scaffolds that are often fragmented due to the presence of a large number of gaps interlaced by repetitive regions. Often in a misassembly different contigs are improperly joined. The contig mis-join problem arises due to inversions, relocation, or a translocation. Gaps arise also due to incorrect insertion or deletion of a particular sequenced read. These changes often result in the wrong placement of a contig onto a scaffold belonging to a different chromosome. Hence, the annotation of unfinished and partially assembled genomes creates ambiguities while accessing complete genetic information as desired by biologists.

Some reasons for incompleteness include: 1. gaps appearing due to polymorphisms in complex genomes where reads on either side of a gap represent two haplotypes that belong to two separate chromosomes, 2. an abundance of repeat elements that confuse the assembler and leave some gaps unfilled, and 3. lack of a sufficient number of reads to cover the part of the genome, requiring an additional library of reads to fill the gaps. Besides, in draft genome assembly base call errors, variations in read coverage depth also cause gaps and pose serious computational challenges while connecting nodes in a *De Bruijn* graph (Guizelini et al., 2016).

Complex eukaryotic genomes are known to contain a large volume of nearly identical copies of DNA repeats and fragments. Various types of repeats present in genomes of wheat, pigeonpea, maize, or potato include transposable elements, highly conserved gene clusters, and segmental duplications. The presence of identical DNA fragments further complicates computational assembly. During pre-assembly, short reads of equal sizes tend to be masked together and complicate the construction of a *De Bruijn* graph (Compeau et al., 2011). Recently introduced third generation single molecule real time technologies (Ardui et al., 2018) and Oxford nanopore technologies (Brown and Clarke, 2016) generate large sized reads which can readily be inserted to fill gaps caused by repetitive elements. Despite virtues, such as low levels of sensitivity and the high sequencing error rates of long read technologies, many plant researchers are opting to use short read sequencing technologies for financial reasons.

Two draft *de novo* genomes compared in the present study are short read assemblies generated from second generation sequencing technologies. Apart from assembly complexity due to smaller reads, repeat abundance also obviates gap closing and is often responsible for the resulting low levels of genome coverage reported in draft assemblies. Modern sequencing platforms generate paired end or mate-pair read libraries. The mate-pair libraries are generated in different sizes and orientations (ranging from 3 to 5 bp and even up to 0.5 kb). They serve as potential inserts while filling gaps. Mate-pair libraries are recommended as a potential approach to mitigate repeats in computational genome assembly (Wetzel et al., 2011; Wang et al., 2012; Grau et al., 2019). In the present work, we demonstrated the application of mate pairs for gap closing during meta-assembly, that resulted in significant improvement of both the genome coverage and quality of the improved pigeonpea assembly.

Major techniques recommended for gaining contiguity and higher coverage in draft genomes broadly include, use of long inserts for gap filling (Wetzel et al., 2011), assembly reconciliation, hybrid assembly (Wang et al., 2012), filtering repeats, and iterative mapping using short reads to close the remaining gaps (Tarailo-Graovac and Chen, 2009; Tsai et al., 2010). The use of paired end or mate pairs for filling the gaps is a robust computational approach. The reconciliation approach (Alhakami et al., 2017) for closing gaps and correcting misassemblies involves comparing available data sets from different draft genomes of the same or related species, mapping their common reads, and finally merging them together to gain improved scaffold lengths with higher contiguity (Kumar et al., 2018; Mishra et al., 2019).

Pigeonpea [*Cajanus cajan* (L.) Millsp.] is a major food legume grown in India and is a diploid (2n = 22) with a genome size of 833.07 Mbp (Varshney et al., 2012). It is a widely cultivated pulse crop and a major source of dietary proteins in India with an annual production of 2.31 mt and productivity of 678 kg/ha (Pande et al., 2013). Prevailing low crop productivity may be attributed to the absence of high yielding cultivated varieties possessing resistance to various pests and diseases. In plants, resistance genes (*R* genes) play important roles in the recognition and protection from invading pests and pathogens. A few sources of resistance to biotic stresses can be found in available germplasm collections. Resistance genes are identified and found primarily organized in individual clusters that are strictly linked across the genome (David et al., 2009). Modern plant breeding techniques include marker-assisted breeding and genomic selection-accelerated development of superior crop varieties with the use of genomic resources and genetic information emitting from sequenced genome projects. The pigeonpea genome was *de novo* sequenced independently by two research groups (Singh et al., 2012; Varshney et al., 2012). These draft assemblies were made available in the public domain (GCA_000340665.1 and GCA_000230855.2), and are valuable resources for breeders. However, both the assemblies are incomplete with a sizable number of fragmented contigs and existing gaps. The lack of accurate genetic information is a major limitation for the prediction of gene compliment components associated with desirable traits. Hence, the primary objective of the present work is to generate a more contagious and complete assembly with improved genome coverage. We report an improved version of a improved assembly based on the genome reconciliation approach that first compares the two available draft assemblies, and scores the matching blocks at each location followed by their merger. The meta-assembler tool employed in the present study detected a significant number of gaps and filled them iteratively using right-sized inserts from local pair-end and mate-pair libraries. The correctness of the mate pairs chosen by the meta-assembler during error correction was further validated by the mapping and alignment algorithm BIMA (Drucker et al., 2014). Completeness and map accuracy of the reconstructed assembly was verified for the presence of conserved plant resistance genes (*R* genes). Here we report the prediction of putative *R* genes, their isolation, and PCR screening of a known resistant cultivar against *Fusarium* wilt disease in both laboratory and field conditions.

## Results

### Improvement of the Draft Genome Assemblies Employing the Reconciliation Algorithm

The reconciliation assembly approach was employed in the present work to refine the incomplete draft genome assemblies, A1 and A2. The assembly tool hybridSPAdes (Antipov et al., 2016) was employed for the selection of optimum k-mers, with evaluated combinations ranging from 21 to 55. We observed that k-mer sizes of 21, 33, 55, and 77 yielded superior assemblies with few fragmented sequences, a smaller number of contigs with high N50, and mean and median scaffold lengths in superior assemblies. The meta-assembler was employed for merging the two assemblies. Merged Illumina HiSeq sequences resulted in 46,979 reads with the N50 length of 24,087. The meta-assembler implemented the reconciliation algorithm to refine and obtain a reconstructed genome. In order to capture the suitable reference assembly set for alignment during the merger process, we examined the required order in which assemblies A1 and A2 were to be chosen as the master set (GCA_000230855.2) and slave sets for alignment with the former (GCA_000340665.1). We observed that choosing A1 as the master set with A2 as the slave set resulted in a highly contiguous superior assembly. The superiority of the merged meta-assembly was systematically evaluated with compression-expansion (CE) statistics. Gaps present in the scaffolds were closed using mate pairs. Gap sizes estimated by the LG_Gapcloser (Xu et al., 2019) were passed on to the next round of alignment. To locate suitably sized inserts, gaps were compared with mate-pair libraries employing BLAST (Altschul et al., 1990) and the single highest scoring mate-pair sequences were chosen. Gap closing mate pairs for gap closing ranged from 200 bp (lower side) to 1,350 bp with 500 bp as the mean size. Mate pairs used by the meta-assembler for gap closing were further validated by mapping and the alignment algorithm BIMA (Drucker et al., 2014).

The remaining gaps were filled by searching unique contig end sequences against unused reads. Analysis of the repeat composition and the identification of their size variations in turn aided the significant reduction of gaps and contributed to the prediction of specific genes. The improved assembly had 46,979 contigs with a total size of 548.2 Mb covering 82.4% of the genome with high contiguity (Table 1).

**TABLE 1:** Genome assembly statistics of draft assemblies A1, A2 and improved A3 assembly.

**Parameter**	**A1, assembly GenBank accession: GCA_000340665.1**	**A2, assembly GenBank accession: GCA_000230855.2**	**A3, improved assembly GenBank accession: GCA_015227855.1**
Number of contigs	360,028	72,923	46,979
Contig N50	5,341	22,480	24,087
Contig L50	30,054	7,524	6,925
Number of scaffolds	NA	36,536	13,101
Scaffold N50	NA	555,764	574,622
Scaffold L50	NA	72	57
Total scaffold length	NA	592,970,700	548,600,000
Number of gaps	NA	72,774	36,561
Range of mate-pair sizes used	NA	NA	20–1,350 bp
Mean size of mate pairs used in gap closure	NA	NA	500 bp
Number of Ns	NA*	34,435,295	34,188,871
Genome coverage	199×	160×	174×
Percentage mapping	75.6%	72.7%	82.4%
GC content	37.2%	32.8%	45.5%
File size (Mb)	648 Mb	605 Mb	548 Mb

Data source: https://www.ncbi.nlm.nih.gov. *“N”s masked.

### Read Mapping

Read mapping increased in the improved meta-assembly from 75.6 to 72.7% in the two compared misassemblies to 82.4% in the improved meta-assembly. A higher number of reads were found to be mapped to the merged assembly compared to those in the A1 and A2 misassemblies. Mapping depth is a measure of the number of reads used for aligning the improved genome. It also helps to estimate the extent of similarity between the improved assembly and the compared misassemblies. Among the two draft assemblies, A2 was superior to A1 in the depth of read coverage. A relatively higher read depth in the A2 misassembly can be attributed to the high-identity Illumina reads used both in the initial assembly and in the later polishing steps. Our final assembly in terms of depth of coverage was superior to A2, with more gaps filled. In addition, the refined assembly had more GC-rich regions (Table 1) with improved gene component predictions. Many gap sequences with high AT composition were eliminated. The total GC content in the A1 and A2 assemblies were 37.2 and 32.8%, respectively and were enhanced to 45.5% in the meta-assembly reported in the present work. The improvement in GC rich fraction and of the N50 values in both contigs and scaffolds in the improved genome were achieved largely due to the application of mate-pair read sequence libraries for gap filling. High GC content is known to be associated with the concentration of coded genes in certain regions in a genome (Rao et al., 2013). In the present study, observed high GC content was obtained in the refined assembly A3 which appeared to be related to an increased number of predicted genes in the improved genome of pigeonpea.

### Meta-Assembly, Annotation, and Quality Assessment

Two draft assemblies were compared, merged, and reassembled employing the two approaches as described above. We initially used unpaired reads for the assembly adopting an overlapping read approach. As no significant improvement was observed in read mapping depth, and eventual coverage, we resorted to available mate paired libraries to close the gaps. We used variable mate pairs during different alignment steps in the meta-assembly and succeeded in resolving repeat problems.

We wanted to ascertain which type of mate-pair libraries effectively resolved the repeat problem. In the assembly, we employed a meta-assembler (Wences and Schatz, 2015). In the first experiment, we only used a 648 Mb library and in the second experiment a 605 Mb and a 548 Mb library were taken together. Initially we used all the single paired read data sets available (minus two mate-pair data sets) of A1 along with all the data sets from A2. In the second treatment, we included two mate-pair data sets from A1 along with all the data available from A2. At the end, all the output values and statistical metrics were collected for comparative performance analysis. We observed that all the available pigeonpea mate-pair libraries taken together resulted in the improvement of genome coverage. It is presumed that the incorporation of variable size mate-pair inserts helped in gap closing during the assembly.

In our final assembly, the contig N50 increased to 24,087, and scaffold N50 increased by 574,622. The total number of gaps decreased across the genome by 50.23% (Table 1). It was observed that the order in which the input draft assemblies were inputted into the meta-assembler drastically influenced the alignment quality and the resulting read coverage (Lindner et al., 2013). In the primary assembly, we treated assembly A1 as the master and aligned it with assembly A2. In the other variant, we used assembly A2 as the master and aligned it against assembly A1. The output resulted in a primary assembly that yielded us a scaffold length of 548,600,000.

### Closure of Repeat-Derived Gaps

For each round of alignment undertaken between the A1 and A2 misassemblies, the meta-assembler built a graph, with vertices of the above alignments and edges joining the two alignments. If both had the same direction, they were readily rearranged into a single block thus providing contiguity. In this case, where the examined genomic segments from two misassemblies did not share the same direction, it indicated that there was an existing distance between them and the prevailing gaps needed to be filled. In such cases, variable size local pair-end and mate-pair libraries could offer right inserts to fill these gaps. While building the graph, the meta-assembler searched the mate-pair library for right sized inserts in order to complete the shortest path between any two of the contigs to fill a gap.

We evaluated the closure performances of the LR Gapcloser (Xu et al., 2019) and Gapfiller (Boetzer and Pirovano, 2012) tools on the repeat-derived gaps. We first tested the performance of each tool using the raw mate-pair reads. Both the above tools used first raw pair-end and mate-pair libraries. We monitored the gap closure efficiency by evaluating the number of gaps closed applying indexed and hashed mate-pair libraries (Drucker et al., 2014). In the merged pigeonpea assembly, we estimated 37,145 repeat-derived gaps of which 584 gaps and 322,780 nucleotides out of a total 34,511,651 were closed. The gap sizes ranged from 20 to 15,510 bp. LR Gapcloser was more efficient in filling most of the gaps, achieving 82.4% and with low error rates.

We achieved improved contiguity by using long mate pairs to fill the gaps in the assembly and thereby achieved higher coverage in the improved assembly. Draft assembly A1 had 360,028 contigs with an N50 and L50 of 5,341 and 30,054, respectively. The reported genome coverage was 199× with a similarity of 75.6%. Draft assembly A2 had 72,923 contigs with an N50 and L50 of 22,480 and 7,254, respectively. A2 had 592,970,700 scaffolds and reported a genome coverage of 160× with a similarity of 72.7%. We presented an improved reference assembly of the pigeonpea genome.

### Completeness of the Merged Assembly

BUSCO (Simao et al., 2015) was employed to evaluate the completeness of the conserved proteins in all three assemblies. The A3 assembly was found to be 94.02% complete. Of the total 1,440 BUSCO groups that were searched, the meta-assembly was found to contain 1,321 complete single-copy (S) BUSCOs, 33 complete duplicated (D) BUSCOs, 57 fragmented (F) BUSCOs, and 29 missing (M) BUSCOs. Whereas comparatively the A1 and A2 assemblies were 85.27% (S:76.87%, D:8.40%, F:5.62%, M:9.09%) and 87.9% (S:80.9%,D:7%,F:5%,M:7.1%) complete, respectively (Supplementary Table 1). The gene completeness score as measured by BUSCO relatively increased in the improved assembly, while the numbers of fragmented and missing BUSCO genes were reduced. This genome comparison can be used to help such draft assemblies toward becoming finished.

### Functional Annotation of Predicted Gene Content

The FGENESH module of the Molquest v.4.5 software package^1^ and Augustus were employed and 51,737 genes were predicted for the improved meta-assembly. The number of predicted genes was less compared to A1 but higher than A2.

In the total gene component prediction, we found 1,303 disease resistance related genes in pigeonpea. The improved assembly yielded a total of 51,737 genes which was less than A1 but more than those reported in the A2 assembly. The variable number of predicted genes observed in the draft assemblies can be attributed to split genes and overestimation during gene finding (Denton et al., 2014). The overestimation of gene numbers often result when fragmented single genes are present on multiple contigs or scaffolds (Pozzi and Salamini, 2007). Improvements in gap filling and read mapping depth resulted in the reduction of the number of genes in meta-assembly A3. The predicted total gene number was less in A3 than in A1 but was slightly higher than the A2 draft assembly (Table 2). In the predicted gene set 54-resistance single copy putative genes containing known conserved domain NBS LRR were selected and *in silico* mapped onto the corresponding chromosomes (Supplementary Table 2).

**TABLE 2:** Repetitive sequences of draft assemblies A1, A2, and the improved A3 assembly.

**Transposable elements**	**A1 assembly**	**A2 assembly**	**A3 assembly**
Retro transposons	77,096,057	116,194,477	89,089,240
Gypsy	52,354,920	71,402,096	59,247,991
Copia	19,937,308	37,676,825	24,339,237
Line	5,261,337	6,717,918	5,914,324
Unclassified elements	216,262,607	169,378,278	158,228,382
DNA transposons	9,772,250	27,455,193	19,826,943
Total transposable elements	303,130,914	313,027,948	267,144,565

### Identification of Repetitive Sequences and Transposable Elements in the Improved Assembly

Repeat elements are extra copies of DNA sequences generated and planted at various locations in the genome to meet certain challenges and improve the fitness of the organism during the course of evolution. Repetitive elements in pigeonpea occupy nearly half of the genome of *Cajanus cajan* (Macas et al., 2015). Repeats pose many computational challenges in read alignment and assembly (Xu et al., 2019), such as the creation of gaps and overlaps and leads to many mapping inaccuracies in misassemblies. One can always filter and exclude the reads but it is essential to map them onto chromosomal locations where gaps exist. Mate-pair libraries were used for resolving repeat problems and obtaining contiguous scaffolds in both prokaryotic (Wetzel et al., 2011) and eukaryotic organisms (Grau et al., 2019). Meta-assembler searches for contigs that can be placed in the gap using mate pairs, and then again checks to see if there exists a recorded shortest path between any of these contigs. In an assembly, overlapping reads are used as edges to connect reads belonging to the same region of the genome. However in complex genomes like pigeonpea, the abundance of repeats cause coverage gaps and read errors thus leaving numerous gaps to fill between contigs while scaffolding. The filling of gaps requires the adoption of robust computational approaches to affectively address repeat problems. Sequenced pair-end and mate-pair reads can potentially bridge gaps efficiently in order to orient contigs by estimating the gap lengths to the edges while filling the scaffolding graph (Ghurye and Pop, 2019).

A high level of assembly was achieved using mate-pair reads in wheat, a genome ridden with a large number of repeats (Clavijo et al., 2017). We analyzed the repeat content in comparison to the A1 and A2 assemblies and divided them into various classes (Table 3). Among the different classes identified, transposable elements were found to be rich in AT elements in A3. In the course of the iterative use of reads during assembly, we observed transposon-derived repeats collapse against identical reads resulting in the closure of significant portions of gaps. Similar observations were reported on gap filling using retro transposon-related repeats in human genome assembly (Marin et al., 2018).

**TABLE 3:** Results of gene search.

**Parameter**	**A1 assembly**	**A2 assembly**	**A3 assembly**
No. of genes predicted	56,888	48,680	51,737
Putative resistance gene analogs against *Fusarium* wilt	NA	NA	54
			

### Identification of Microsatellites

The improved pigeonpea assembly was mined for simple sequence repeats (SSR). A total of 297,294 were simple repeats out of the total 298,732 repeats and the remaining 1,438 belonged to complex types (Table 4). Mononucleotide repeats were abundant with 56.05% of the total SSRs mined. Dinucleotides occupied 33.45% (99,949), 8.72% (26,069) were trinucleotides, and 1.27% were tetranucleotides (3811) repeats. The remaining SSRs were of the complex type, 0.25% were hexa nucleotides and 0.22% were penta.

**TABLE 4:** Results of microsatellite search in the improved pigeonpea assembly A3.

Total number of sequences examined	13,101
Total size of examined sequences (bp)	584,435,790
Total number of identified SSRs	298,732
Number of SSR containing sequences	6,494
Number of sequences containing more than 1 SSR	4,603
Number of SSRs present in compound formation	41,002

**FIGURE 1:**
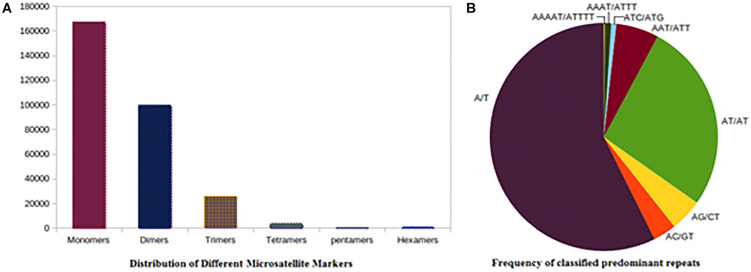
SSR distribution frequency. **(A)** Distribution of different repeats type classes. **(B)** Frequency of classified predominant repeats.

Among the 167,465 mononucleotide repeats, the mononucleotide motifs were in majority with A/T repeats of 98.25 and 1.74% were occupied by C/G types. Among the 99,949 dinucleotides microsatellites, the AT/AT type (77.34%) of microsatellites were most common in the genome followed by the AG/CT type (13.21%), and the AC/GT type (9.40%). The CG/CG type dinucleotides microsatellites were present in a very low proportion (0.03%). In trinucleotide SSRs repeats (26,069), around 66.71, 12.31, 8.07, and 5.98% of SSRs were of AAT/AAT, AAG/CTT, ATC/ATG, and AAC/GTT types, and were most abundant, respectively. Among the other types of repeats, the ACG/CGT type was lowest (0.36%) in the genome of pigeonpea. The highest distribution (68.06%) of tetra nucleotides microsatellites was present in the genome of pigeonpea. The maximum numbers of predominant SSRs repeats were of the A/T type followed by AT/AT, AG/CT, AAG/CTT, AAT/ATT, and AAAT/ATTT (Supplementary Table 3). The overall analysis showed that the relative abundance of tetra, penta, and hexa SSRs types were low as compared to mono, di, and tri SSRs types in pigeonpea genome sequences (Figure 1).

### Characterization and Synteny Analysis of Pigeonpea NBS-LRR Like Resistance Gene Analogs

We verified the presence of already known conserved disease resistance gene families in the refined meta-assembly. The reported resistance (R) genes containing nucleotide-binding site (NBS)-leucine rich repeat (LRR) protein sequences from other important legume genomes were downloaded from Phytozome (Goodstein et al., 2012). The comparison of the predicted coding sequences against bean (*Phaseolus vulgaris*) clusters resulted in the identification of more than 100 resistance gene analogs (RGA). An annotation of the mined predicted genes revealed the presence of known disease resistance domains, such as ARC-NBS-LRR, transmembranes, and kinases. Nucleotide-binding sites (NBS) containing disease resistance genes play an important role in defending plants from a variety of pathogens and insect pests. Many R genes have been identified in various plant species including the pigeonpea genome (Singh et al., 2012; Varshney et al., 2012). However, functional R genes targeting specific diseases in pigeonpea have not been reported. In this study, an improved A3 meta-assembly using computational analysis of the refined genome identified NBS-LRR resistance (R) proteins. The 1,301 mined putative resistance gene analogs were shown to share up to 78% of their homology with soybean, chickpea, barrel clover, field bean, and other species (Supplementary Table 4). Of them, 251 NBS-LRR domains containing pigeonpea resistance gene analogs were selected. The RGAs had a high amino acid identity in the identified putative pigeonpea disease resistance genes, which showed a high level of proteins in *Glycine max* with several sequences with high homology up to (77–98%) (Supplementary Table 5). We identified 54 NBS-encoding single copy genes and characterized them on the basis of structural diversity and conserved protein motifs.

**FIGURE 2:**
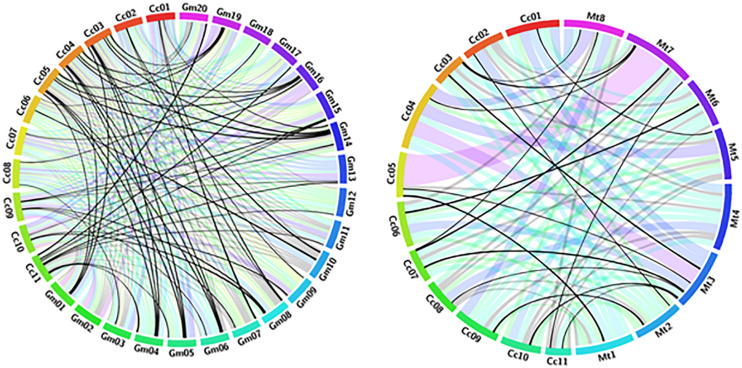
Circos diagram presenting syntenic relationships between NBS-LRR containing R gene proteins in pigeonpea (Cc), *Glycin max* (Gm), and *Medicago truncatula* (Mt) pseudomolecules. Pseudomolecules of the two target species were labeled as Gm01-20 and Mt1–8. Pigeonpea pseudomolecules are labeled in different colors and labeled as Cc01-11. Collinear blocks are colored according to the color of the corresponding pigeonpea pseudomolecules. Each ribbon radiating block from a pigeonpea pseudomolecule represents a NBS-LRR similarity block between pigeonpea and other legumes.

Synteny analysis revealed significant relationships among the selected legume genomes. *Glycine max* and *Medicago truncatula* genomes revealed the presence of a high level of extensively conserved regions among pigeonpea and other legumes. We observed that nearly 89–91% of the pigeonpea assembly showed significant signs of RGA conservation with other legumes, viz., 41 NBS-LRR orthologs in *Glycin max* and 73 NBS-LRR orthologs in *Medicago truncatula.* A total of 57% of NBS-LRR pigeonpea genes were identified for the closely related organisms. *Glycine max* was found to share the largest number of extended conserved syntenic blocks with *Cajanus cajan* indicating its recent ancestry, followed by *Medicago truncatula*. The reported A3 meta-assembly of pigeonpea comprises 251 R gene homologs of the disease resistance gene, of which 229 are anchored to different pseudomolecules of pigeonpea. Of these, 23 genes are distributed to 57 collinear blocks between pigeonpea and the Glycine max genomes displaying a high level of collinearity (Figure 2). Overall, all pigeonpea RGAs displayed extensive collinearity with the different chromosomes of Glycine max and *Medicago truncatula*. Synteny analysis revealed homologous blocks connecting chr4 in *C. cajan* with chr4 of *G. max*; chr11 of *C. cajan* with chr20 and chr17 of *G. max*; and chr3 of *C. cajan* with chr19 in *G. max.* Similarly, comparative analysis reported in draft assembly A2 (Varshney et al., 2012) confirms the presence of homologous blocks connecting chr3 in *C. cajan* with chr19 of *G. max* (Varshney et al., 2012).

### Cloning, Isolation, and PCR Amplification of Identified Putative R Gene Analogs (RGAs)

The genomic DNA samples from 25 known pigeonpea cultivars were scanned for presence of identified putative R genes. EPrimer (Spapé et al., 2014) was employed for designing the PCR primer sets. A list of the primer sequences used in PCR amplification are given in Supplementary Table 7. Eluted PCR amplificons were sequenced by the Sanger sequencing method. Isolated pigeonpea resistance gene analogs were deposited to NCBI (Supplementary Table 6).

## Discussion

In the present work, we chose two available incomplete draft assemblies and employed a reconciliation algorithm to correct any errors. The two compared draft assemblies A1 and A2 had low genome coverage with several repeats and gaps causing disjoints between contigs. A meta-assembler was employed in the present work based on the genome reconciliation algorithm. The computational framework included a merger between the two draft assemblies, A1 and A2, aligning them by selecting common homologous sequence matches and mismatches present in both, resolving gaps, and other sequence errors, to obtain a consensus and complete assembly.

To begin with we wanted to select the order in which the input draft assemblies were to be merged to gain a subsequent superior alignment with higher read depth and read mapping. After several permutations, we observed that treating assembly A1 as the master and aligning it with assembly A2 yielded better read mapping and lengthier scaffolds of 592,970,700 mb. Merging the two draft assemblies, in course of alignment, the meta-assembler yielded matched and mismatched portions in the merged assembly by identifying homologous genomic regions with a shared set of reads. Mismatches included gaps that had to be filled with right sized read sequences.

The meta-assembler initially utilized all available raw reads from both draft assemblies using conventional read overlapping techniques to fill the existing gaps and join the contigs. However, no notable success was observed in gap filling and repeat resolution. Alternatively, we employed local pigeonpea pair-end and mate-pair libraries to fill the gaps. The meta-assembler generated statistics comparing the distances between the mapped mates and the required sizes of insert reads to fill a gap. For example, gaps measuring <500 mb were filled by pair-end reads while mate-pair reads were utilized for filling gaps measuring 3–5 KB. Mate-pair sizes selected by the meta-assembler were further compared and validated using indexed and hashed mate libraries employing the alignment tool BIMA (Drucker et al., 2014). There are reports on the use of large sized mate-pairs for filling bigger gaps in assembly (Potato Genome Sequencing Consortium, 2011). In the present study, we employed pair-end and mate-pair reads which contributed significantly to filling gaps and thereby in joining the contigs to the full length scaffolds. Further, iterative use of pair-end and mate-pair libraries during successive alignments resulted in the identification of maximal portions shared by the same library of reads. This in turn contributed to the dramatic improvement of genome coverage in the resultant A3 assembly. The quality of the A3 assembly was judged using metrics—contig number, scaffold lengths, N50 and L50, and genome coverage of 160× with a similarity of 72.7%. The genome similarity score can also be used in estimating the extent of redundancy present in both genomes.

Draft assembly A1 had 360,028 contigs with an N50 and L50 of 5,341 and 30,054, respectively. We obtained genome coverage of 199× with a similarity of 75.6%. Draft assembly A2 had 72,923 contigs with an N50 and L50 of 22,480 and 7,254, respectively. A2 had 592,970,700 scaffolds with a reported genome coverage of 160× with a similarity of 72.7%.

**FIGURE 3:**
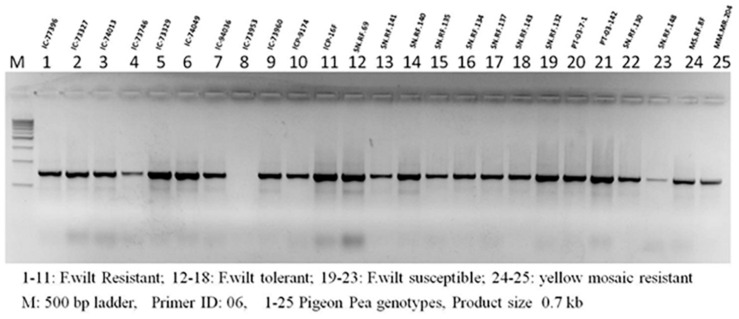
PCR amplification of resistant gene analog from pigeonpea germplasm accessions with differential resistance reaction for Fusarium wilt disease. Field evaluation of 25 accessions at two locations in two years showed 14, 6 and 5 accessions as resistant, tolerant and susceptible respectively (Supplementary Table 8).

FGENESH predicted 51,737 genes using the improved meta-assembly. The predicted number of genes was less in our improved assembly (Supplementary Table 2) compared to A1 but was higher than A2 (Table 2). An annotation of the improved assembly yielded 51,737 predicted genes. Wet lab PCR amplification is the gold standard for verifying predicted gene presence and their functionality. For PCR-based gene amplification, 23 primer sets were designed to screen 34 pigeonpea cultivars. Out of the 34 genotypes screened, 14 were found to be *Fusarium* wilt resistant (Supplementary Table 8), 6 were *F*. wilt tolerant, 5 were *F*. wilt susceptible, and 5 had yellow mosaic susceptible genotypes (Figure 3). Data on yellow mosaic disease reaction are not presented here. PCR amplified genes were isolated, cloned, and submitted to NCBI (Supplementary Table 6). Genotype environment interaction in the field determines the phenotypic performance of isolated plant genes. The phenotypic evaluation of predicted resistance genes in field trials is also required for the transfer of obtained results to pigeonpea downstream breeding programs for the development of disease resistant cultivars. Field experiments were conducted to assess the disease reaction of the predicted R genes to *Fusarium* wilt taking cv. Asha (object of the present study) as control with 25 pigeonpea cultivars. The replicated field experiments were conducted at Ranchi (Jharkhand state) and Rahuri (Maharashtra state), India during the rainy season of 2011 and 2012. Of the 25 cultivars screened along with check *cv*. Asha, 14 resistant and six tolerant disease reactions at the Ranchi farm and eight resistant, one tolerant, and six susceptible disease reactions at the Rahuri farm were observed for the F. wilt disease of pigeonpea. The observed variation in disease incidence reflects the natural agro-climatic conditions prevailing at the individual trial sites.

## Conclusion

In the present work, a genome reconciliation algorithm was adopted to merge and reconstruct draft assemblies to produce an accurate and near complete genome assembly of pigeonpea. We demonstrated the successful implementation of our reassembly framework by merging two chosen draft assemblies employing pair-end and mate-pair libraries to correct gaps and other sequencing errors. The resulting reconstructed meta-assembly was superior compared to the two draft assemblies in terms of measured assembly quality statistics, *viz*., N50 and scaffold lengths. The quality of the improved assembly was assessed for the presence of known conserved resistance gene loci (imparting resistance to *Fusarium* wilt disease in pigeonpea). An annotation of the improved assembly yielded a prediction of 1,303 resistance genes (including six extra genes gained from the meta-assembly). PCR screens and field experiments validated the resistance reaction of isolated genes against *Fusarium* wilt thus making the results available to pigeonpea breeders.

## Materials and Methods

**FIGURE 4:**
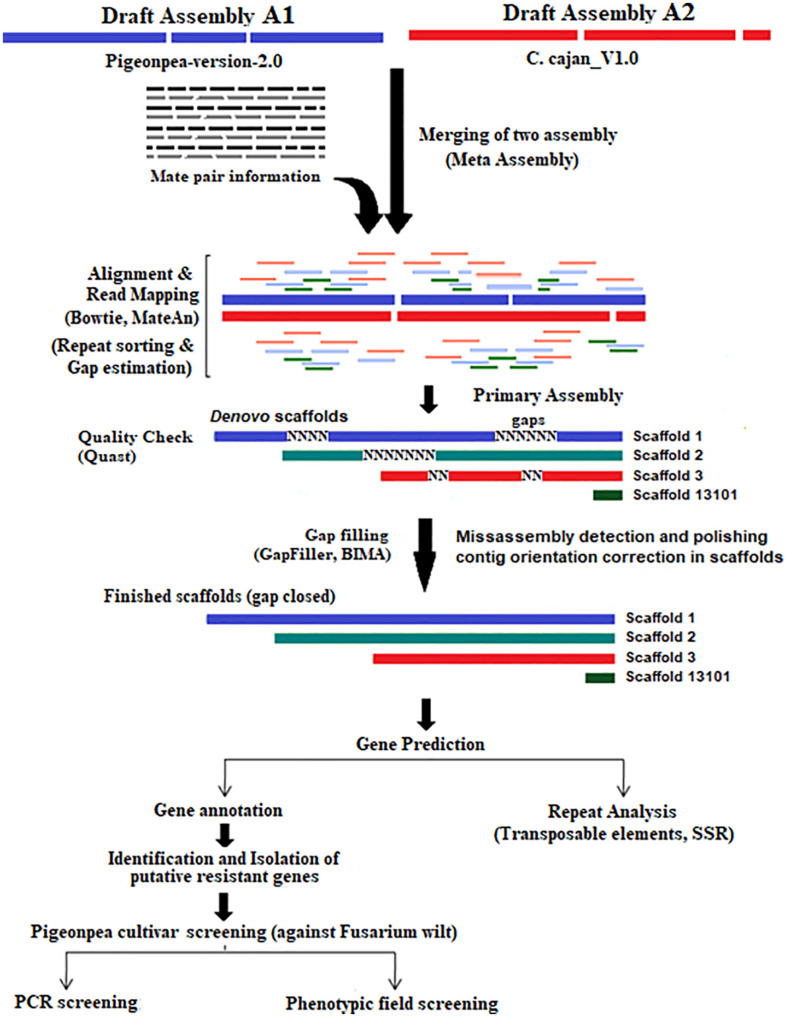
Experimental framework depicting the reconstruction steps of the pigeonpea genome.

We developed a workflow model (Figure 4) based on a reconciliation algorithm, that includes: 1. A merger of the two misassemblies, 2. finding matches and mismatches and other sequencing errors, 3. gap closing using pair-ends and mate-pair libraries, and 4. the assessment of improved assembly quality, and the prediction, isolation, and characterization of disease resistance gene families.

### Retrieval of Pigeonpea Genome Datasets

Complete data sets belonging to two whole genome sequences of pigeonpea and the associated 23 SRA reads were downloaded from the National Center for Biotechnology Information (NCBI)^2^ to local storage—GCA_000340665.1 (SRA accessions SRR5922904-SRR5922907) and GCA_000230855.2 (SRA accessions SRR6189003-SRR6189021) for the *cv* Asha.

### PCR Amplification

Genomic DNA from 15-day-old seedlings of 34 pigeonpea cultivars was extracted employing the CTAB method. The purity and concentration of DNA was estimated with Nanodrops ND-1000. Nine primers were selected for the polymorphism study (Supplementary Table 7). Polymerase chain reaction (PCR) was performed on a total volume of 20 μl containing 60 ng of template DNA, 200 μM of dNTPs, 2.5 mM of MgCl2, 1× PCR buffer, 0.4 μM of each primer, 0.75 U of Taq DNA polymerase, and water to make the final volume up to 20 μl. For designing the primer sets for the PCR amplification of predicted resistance gene (R) orthologs, BLASTN was employed against the soybean genome. EPrimer (Goodstein et al., 2012) was employed for designing PCR primer sets. A list of primer sequences used in PCR amplification are given in Supplementary Table 7. Eluted PCR amplificons were sequenced by the Sanger sequencing method.

Amplifications were carried out using the Bioer Gene Pro thermocycler and PCR conditions were set as an initial denaturation at 94°C for 5 min, 30 cycles of denaturation at 94°C for 30 s, primer annealing at 50°C for 30 s, primer extension at 72°C for 2 min, and a final extension step at 72°C for 7 min. The amplified products were visualized by ethidium bromide-stained 1.5% agarose gels in a SYNGENE G-Box gel documentation unit (Figure 3).

### Genome Reconstruction and Quality Assessment

Illumina pair-end and mate-pair library sequence reads of pigeonpea and *cv* Asha were quality checked using FASTQC v0.11.8^3^. Contaminated reads were removed to obtain error-corrected reads. Reads with sequence quality Phred scores of less than Q30 (base calling accuracy with less than 99.99%) were removed using PRINSEQ v0.20.4^4^ and reads were repaired using BBmap v37.66^5^.

Reported pigeonpea draft assemblies A1 (Singh et al., 2012) and A2 (Varshney et al., 2012) were both sequenced using Illumina technology and assembled with the SoapDenovo v2.3.1 assembler. In the present work, data sets A1 (GCA_000340665.1 consisting of 4 SRA read sets) and A2 (GCA_000230855.2 of 19 SRA read sets) were analyzed employing a reconciliation algorithm (Tarailo-Graovac and Chen, 2009). The work flow included the steps: (1) the merger of the two misassemblies, (2) finding matches, mismatches, and other structural errors, (3) closing gaps using pair-end and mate pair libraries, (4) the validation of mate-pair sizes used in the meta-assembly using indexed and hashed mate-pair library sets, (5) an assessment of the quality of the improved assembly, and (6) a prediction of disease resistance gene families, their isolation, and characterization.

A1 consisted of 360,028 initial contigs (N50 5341, 648 Mb) with 30% of gaps within contigs. A2 contained 72,923 scaffolds (N50 22480, 605 Mb) with 20% intra scaffold gaps. We used all the read datasets available belonging to A1 and A2 with NCBI. All the computations including read pre-processing, quality control, comparison of the two draft assemblies, their alignment, gap filling, assembly merger, map accuracy, quality assessment, and putative gene prediction were performed on a HPC server employing a meta-assembler (Wences and Schatz, 2015).

LG_Gapcloser and GapFiller (Boetzer and Pirovano, 2012; Xu et al., 2019) were employed to find the existing gaps (A1 30%; A2 20%). Mate-pair libraries were hashed and gap sizes were validated using the alignment tool BIMA (Drucker et al., 2014). Initially short reads were used for filling gaps, resulting in a genome size of 648 Mb in A1 and 605 Mb in the A2 draft assembly.

Draft assembly A1 was sequenced in 2011 and had a genome coverage of 199× (Singh et al., 2012). However, using the same raw read data, assembly A3 reported a gain of coverage, i.e., an increase of ∼15% (from 60.0 to 75.6%), and was then resubmitted to NCBI. In our present work, we used this recent assembly set and A1 and A2 assembly data (Varshney et al., 2012) for reassembly and improvement (Figure 4).

We observed that in our reassembly, pair-end insert read sizes below 500 bp in our library were utilized for filling smaller gaps. Although mate-pair sizes up to 5.0 kb are available in our library, a 1,350 kb size was the largest used insert. In our meta-assembly, these mate pairs were employed affectively for closing medium and long-distanced gaps (even up to 20–25 kb). Similar results on the use of large sized mate-pairs for filling bigger gaps was reported in the assembly of large genomes (Ghurye and Pop, 2019).

### Merging Misassemblies and Gap Closure

Draft assembly sequences A1 and A2 were merged into a single sequence. The alignment and merger of the A1 and A2 assemblies resulted in a total scaffold length of 548 Mb. The resulting merged assembly was compared to the A1 and A2 draft assemblies (75.6 and 72.7%, respectively) and had an improved genome coverage of 82.4%. Yet the merged sequence contained 10% of gaps.

To improve further contiguity and accuracy of the merged sequence, existing intra scaffold gaps were filled. Repeat content and existing gaps were estimated by Gapcloser (Xu et al., 2019) and Gapfiller (Boetzer and Pirovano, 2012). In the second round of gap filling, various computational approaches, such as paired end, mate-pair libraries, and remaining unused short reads were used. The gap content and the estimation of repeats is shown in Table 1. Iterative use of the leftover short reads (300 bp) contributed to filling nearly 20% of the gaps. After polishing and another round of reassembly, a scaffold length of 13,348 (scaffolds of N50 574,622) with a coverage of 174× was yielded.

### Improved Genome Assembly and Quality Assessment

Increased N50, maximum scaffold length and minimum number of contigs, increased N50 values together with longer scaffolds contributed to improving the genome coverage. In the misassemblies, the number of gaps and Ns caused by repeats were measured. In the course of meta-assembly, we strived to minimize gaps and other sequencing errors. We employed Quast v4.5 (Gurevich et al., 2013) to gather extensive assembly statistics. BUSCO v3.2 (Simao et al., 2015) was employed for assessing the genome completeness, and the annotation and sets of predicted genes. Mapping accuracy and the identification of resistant gene analog loci were assessed (Supplementary Table 1). In addition, 75% of unigenes were aligned to the reassembled genome.

### Gene Prediction and Function Annotation

The meta-assembly was first repeat-masked using the Repeat Modler and Repeat Masker tools (Smit et al., 2013), followed by *ab initio* gene prediction using the FGENESH module of the Molquest v4.5 software package^6^. The predicted genes were annotated using the BLASTX (*E* < 10^6^) search against the NCBI non-redundant (nr) protein database using the Blast2GO software (Conesa et al., 2005). Synteny blocks between the genomes of pigeonpea and other legumes were computed by blastp combined with the Circos (Krzywinski et al., 2009) to understand homology to the NBS-LRR gene from *Glycin max* (Gm) and *Medicago truncatula* (Mt) pseudomolecules.

### Identification of Genome Wide SSR

The refined genome sequence of pigeonpea was analyzed to identify various simple sequence repeats (SSR) types using the Microsatellite Identification tool (MISA)^7^. The minimum length for SSR motifs per unit size was set to 10 for mono, 6 for di, and 5 for a tri, tetra, penta, and hexa motifs. We calculated the total lengths of all mono-, di-, tri-, tetra-, penta-, and hexa-nucleotide repeats in terms of base pairs of SSR per mega base pair (Mb) of DNA.

### Gene Validation

The genome similarity score recorded a set of sequenced reads originating from one draft genome correctly mapped onto a second genome. To check the accuracy in the improved pigeonpea genome, we wanted to verify the location of certain genomic regions or loci present in the inputted two assemblies. A set of genes imparting resistance against various pests and diseases were located in the B4 cluster on chromosomes in the two examined draft assemblies of pigeonpea (*Cajanus cajan*) Asha. As a test case, the location of B4 gene cluster syntenic regions were verified in the present study to estimate the accuracy of read mapping achieved in the improved assembly.

### Computational Resources

We ran all reassembly and merging operations using HPC Cluster with CentOS-Linux version 7,2.93 GHz 2× Intel Xeon 8 core processors and 2 TB of RAM. The majority of the running time was spent on the assembly process and about 1/4 of the time was spent on graph construction and analysis. However, Reconciliator used more than 1.5 TB of RAM to merge the pigeonpea draft assemblies.

## Data Availability Statement

The improved draft genome assembly of Pigeonpea is available at NCBI GenBank, under the Accession Number GCA_015227855.1. All datasets generated for this study are included in the article/Supplementary Material, further inquiries can be directed to the corresponding author.

## Author Contributions

SM conceptualized and supervised all the experiments, interpreted the results, and wrote the manuscript. PM and RM performed all the high-throughput bioinformatics analysis, interpreted the results, and formulated the manuscript. MS conducted field phenotyping experiments. DW, AK, NS, and RK updated the final manuscript for publication. MY and SS performed all the wet lab experiments. All authors contributed to the article and approved the submitted version.

## Conflict of Interest

The authors declare that the research was conducted in the absence of any commercial or financial relationships that could be construed as a potential conflict of interest.

## Footnotes

1 http://www.softberry.com

2 https://www.ncbi.nlm.nih.gov

3 http://www.bioinformatics.babraham.ac.uk/projects/fastqc

4 https://sourceforge.net/projects/prinseq

5 https://sourceforge.net/projects/bbmap

6 http://www.softberry.com

7 http://pgrc.ipkgatersleben.de/misa/

## Supplementary Material

Supplementary Table 1 BUSCO (Benchmarking Universal Single-Copy Orthologs) genes distribution.

Supplementary Table 2 Putative disease resistance genes predicted from the improved reassembly of pigeonpea.

Supplementary Table 3 Numbers of predominant SSRs.

Supplementary Table 4 Resistance gene analogs sharing homology with species.

Supplementary Table 5 NBS-LRR domain containing resistance gene analogs to pigeonpea [*Cajanus cajan* (L.) Millsp.].

Supplementary Table 6 List of pigeonpea disease resistance genes submitted to NCBI.

Supplementary Table 7 List of primer sequences used in PCR amplification.

Supplementary Table 8 Pigeonpea phenotypic field scores for *Fusarium* wilt disease reaction.
