# Supplementary material for: Refinement of Draft Genome Assemblies of Pigeonpea (Cajanus cajan)
Source: Front Genet. 2020 Dec 15;11:607432. doi: 10.3389/fgene.2020.607432 (PMC7770131; doi:10.3389/fgene.2020.607432)
Supplement: Supplementary Table 1 — BUSCO (Benchmarking Universal Single-Copy Orthologs) genes distribution. [file Table_1.DOCX]

Supplementary File 6 : List of primer sequences used in PCR amplication.

>AFSP01001376.1_contig01377.2 FGENESH:[mRNA] 2 2 exon (s) 5236 - 6259 393 bp, chain + ATGGATCATGGCACTTCAATAGGTACCAGTGAAGACGGGATGGGTAAGACTAAACTTGTC CAACATTGTGTATACAATGACCCAAAGATGAAGGAAGTTAAATTTCATATCAAAGCTCGG GTTTGTGTTTCAAATGATTTTGATGTTTTGACATTTGCAAGAGCTATTCTTAAGGCAATT ACTAAATCAAAAGATGATGGTGGAGACCTAGAAATGGCTAATGGAAGATTGAAAGAAAAG TTGTTAGGAAAGAGTTTTCTTATTGTTTTAGATGATGCTTGGAATGAAAGGCAAGAGAAA TGGGAATTTATGGAAACTCCTATTATTTATAAGGCTTGGGGAAGTATAATTCTCGTCACA ACATGTGGTAAGAAAGTTATTTCTACCATGTGA

>AFSP01001376.1_contig01377.2 FGENESH: 2 2 exon (s) 5236 - 6259 130 aa, chain + Domain - NB-ARC MDHGTSIGTSEDGMGKTKLVQHCVYNDPKMKEVKFHIKARVCVSNDFDVLTFARAILKAI TKSKDDGGDLEMANGRLKEKLLGKSFLIVLDDAWNERQEKWEFMETPIIYKAWGSIILVT TCGKKVISTM

Primer3 output

OLIGO start len tm gc% any 3' seq

LEFT PRIMER 111 20 60.15 45.00 6.00 0.00 CAAAGCTCGGGTTTGTGTTT

RIGHT PRIMER 304 20 60.18 45.00 2.00 1.00 CCCATTTCTCTTGCCTTTCA

SEQUENCE SIZE: 393

PRODUCT SIZE: 194 #########################################################################################

>AFSP01002013.1_contig02015.3 FGENESH:[mRNA] 3 4 exon (s) 5059 - 6672 672 bp, chain + ATGGTTGGACAAAGTGCAATGTTGATGGATATTTTTTTTATGGGGGATTCTGAGTGCGCT GCATGTGATTTCAGAATTCACACATTGTCGCATTTAACTGTTCAAAACTATTACTTGAAG ATGCGAGAATCACATTTTATCTTGGTTGCATTTGACAGGCTGGCTTCTCAAGTTTTAGAT TTAATTCATGTAAGAAAACTTGGTGAGGAGCTGCTGAAAAAGTTGAACATCAAGCTGTTG TCTATTGATGCTCTCGCTGATGGTGCTGAACAAAAGCAGTTCAGAGATCCACTTGTGAAA GCATGGCTTACTGCGGTCAAAGATGCTGTGTTTGATGCAGAGGATCTCCCGGATGAAATA GACTATGAAATCTCCAAGCCTTTACTTACAAGCAAAGTTGGCTTGGGGATGAAACAAGTC CTTGACAAACTAGAACATCTTGCAAGCCAAAAAGGTGATCTTGGTTTGAAAGAGGCTACT TATTCTGGTATTGGATCAGTTTTTCCTAAACATGCATTCCAAGATGGTCACCCTCGGTTG ATTGTTGAGTTGAAGGAGATTGTTACAAAGATAGTTGAAAAATCCAAAGGATTACCTATG ACCTTGAAAACAATTGGAAGTCTTTTACATACAAAGTCATTGTGGGTTATTGAAGCTGCA GCAGAAGAGTGA

>AFSP01002013.1_contig02015.3 FGENESH: 3 4 exon (s) 5059 - 6672 223 aa, chain + Domain - NB-ARC, NBS_ RR

MVGQSAMLMDIFFMGDSECAACDFRIHTLSHLTVQNYYLKMRESHFILVAFDRLASQVLD LIHVRKLGEELLKKLNIKLLSIDALADGAEQKQFRDPLVKAWLTAVKDAVFDAEDLPDEI DYEISKPLLTSKVGLGMKQVLDKLEHLASQKGDLGLKEATYSGIGSVFPKHAFQDGHPRL IVELKEIVTKIVEKSKGLPMTLKTIGSLLHTKSLWVIEAAAEE

OLIGO start len tm gc% any 3' seq

LEFT PRIMER 325 20 59.99 50.00 4.00 0.00 GCTGTGTTTGATGCAGAGGA

RIGHT PRIMER 534 20 59.78 50.00 5.00 1.00 AGGGTGACCATCTTGGAATG

SEQUENCE SIZE: 672

PRODUCT SIZE: 210

#########################################################################################

>AFSP01029727.1_Contig29789.1 FGENESH:[mRNA] 1 1 exon (s) 7031 - 9070 2040 bp, chain - ATGGCAGTAGCATTAGTTGGTGGTGCTCTTCTTTCTGCTTTCCTCCAGGTTGCATTTGAC AGACTTGCTTCTCGTCAAGTTTTGGACTTCTTTCTTGGAAGAAAGCTCGATGAGACACTG GTGAGCAACTTGAACACCATGCTGCGGTGCATCAATGCTTATGCTGAGGATGCAGAACAA AAGCAGGTCAGAGATTCACGCGTGAAAGCATGGCTTACTGATGTCAAAGATGTTGTGCTT GACGCAGAGGACCTCTTGGATGAAATAGACTATGAACTCTCAAAAGCTGAATCTGAATCT CAAACCTGTACTTGCAAGGTAACAGTACCTTTCTTTAATGCTGCTTTAAGTTCATTTAAC AGGAAAATTGAGTCAAGGATGAGACAAATCCTTGAAAAATTGGAATATCTTGCTGGGTTA AAGGGTGATTTGGGTTTGAAAGAGTCTACTGATTCTGGTGTTGGATCAGTTAGTGAAGTG TTACAAAGGGTGCCATCAACGTCTTTGCTTGGTGGAAGTGTTTTATATGGTAGAAATGAT GACAAAGAAGTGATTTTTAATTGGCTGATGTCTGACATTGAGAATGGTAACCATCCATCA GTATTTTCTATTGTGGGTATGGGTGGGATGGGTAAGACCACGCTTATGCAACATGTATAC AATGATTCAAAGATAGAGGGTAAATTTGATATTAAAGCTTGGGTTTATGTTTCAGATGAT TTTGATGTTTTAAAGGTATCAAGAGCAATTCTTGACACAATTACTAAATCAGTTGATGAT AGTAGAGAGCTAGAAATGGTTCATGGAAGATTGAGAGACTTTTTGACAGGAAAGAGATTT CTTCTTGTTTTGGATGATGTTTGGAACAAAAAACAAAAACAATGGGAAGCTTTGCAAACT CCTCTTAGTTATGGAGCTCAAGGAAGTAAAATTGTTGTCACCACACGTGACATGAAAGTT GCTTCAACTGTCAGATCAAACAAGATACATCTCCTAAGACAATTACAAGATGATCATTGC TGGCAATTGTTTGCTAAACATGCATTCCATGATGAAAATCCTCAATCAAATTCACACTGC AAGGCAATTGGTATGAAGATAGTTGAAAAATGCAAAGGACTGCCACTAGCCTTGTCAACA ATTGGAGGTTTGTTACACAAAAAATCATCTATTTTGCAATGGGAGAGTGTATTAACAAGT GAGATATGGGAATTTTCTGAAGAGGATAGTGAAATTATCCCTGCTTTATTATTAAGCTAT CACCACCTTCCTGCTCAACTCAAGAGATGTTTTGCTTATTGTGTCTTATTTCCCAAAGGT TATGAGTTTGACAAGGAGGATTTAGTTCTGTTATGGATGGCTGAAAATTTTGTACAGTGC TCTCAACAGAATATGAGTATGTTAGAAGTTGGCAGACAATACTTTGATGATCTGCTATCA AGGTCCTTTTTTCAACAATCAGGTGGAGAGCAGATGTATTATGTCATGCATGATCTTCTC AATGATTTGGCAAAATATGTTGGTGGGGATTTTTGTTTCAGGTTGGACGTTAAAGAAACA ACAATTATTCCTATAACCACCCGCCATTTTTCATTGGGACAGAATTGCATTCATAATTTT GCAGGGTTTAAGGGTTTATATCATGGTGACAAATTGCGGACATTTCTACCATTAAGTACG ACTCCAAATGGTATTGTATGTTGGAGTTGTAGAACATCTAGCACGTTCATAAATGATTTG TTTTTGAAGTTTAAGTACTTGCGTGTCTTATCTTTGTCGGGTAATTCTAGTCTTACAGAG GTACCTGACTCTATAGGCAATCTTAAACATCTTCGTTCTCTCGACCTTTCTTGTACCCAT ATAAGAAAGTTGCCTGATTCAATATGTTCACTCTATAATTTACAAATACTGAAGCTGAGA TATTGTGCACATTTTGGAGGGCTGCCCTTGAACTTTAATAAACTCAAATTGCAACTCCTT GATTTGTCGGGAACCGATGTAAATAAGACGGCAATGACTTTAGTATCTAGATCCCGATAA

>AFSP01029727.1_Contig29789.1 FGENESH: 1 1 exon (s) 7031 - 9070 679 aa, chain - Domain - NB_ARC, NBS-

RR, signal peptide MAVALVGGALLSAFLQVAFDRLASRQVLDFFLGRKLDETLVSNLNTMLRCINAYAEDAEQ KQVRDSRVKAWLTDVKDVVLDAEDLLDEIDYELSKAESESQTCTCKVTVPFFNAALSSFN RKIESRMRQILEKLEYLAGLKGDLGLKESTDSGVGSVSEVLQRVPSTSLLGGSVLYGRND DKEVIFNWLMSDIENGNHPSVFSIVGMGGMGKTTLMQHVYNDSKIEGKFDIKAWVYVSDD FDVLKVSRAILDTITKSVDDSRELEMVHGRLRDFLTGKRFLLVLDDVWNKKQKQWEALQT PLSYGAQGSKIVVTTRDMKVASTVRSNKIHLLRQLQDDHCWQLFAKHAFHDENPQSNSHC KAIGMKIVEKCKGLPLALSTIGGLLHKKSSILQWESVLTSEIWEFSEEDSEIIPALLLSY

HHLPAQLKRCFAYCVLFPKGYEFDKEDLVLLWMAENFVQCSQQNMSMLEVGRQYFDDLLS RSFFQQSGGEQMYYVMHDLLNDLAKYVGGDFCFRLDVKETTIIPITTRHFSLGQNCIHNF AGFKGLYHGDKLRTFLPLSTTPNGIVCWSCRTSSTFINDLFLKFKYLRVLSLSGNSSLTE

VPDSIGNLKHLRSLDLSCTHIRKLPDSICSLYNLQILKLRYCAHFGGLPLNFNKLKLQLL DLSGTDVNKTAMTLVSRSR

OLIGO start len tm gc% any 3' seq

LEFT PRIMER 479 20 59.96 45.00 5.00 1.00 TGTTACAAAGGGTGCCATCA

RIGHT PRIMER 637 20 60.01 50.00 2.00 2.00 TCTTACCCATCCCACCCATA

SEQUENCE SIZE: 2040

PRODUCT SIZE: 159 ####################################################################################

>AFSP01054890.1_contig55006 FGENESH:[mRNA] 2 1 exon (s) 1402 - 2013 612 bp, chain + ATGCCAATGCATTTGGGAAGACTAAAAACTCTTCAAGTATTGAGTTCGTTTTATGTTGGC AAAAGTTGCGAGTTCAATATTAATCAACTAAAAGGACTCAATCTTCATGGAGGGCTATCA ATTGGGGAGCTGCAGAATATTGTGAATCCGGCAGATGCATTTGCTGCGAGTCTTAAAAAT AAAACACACCTTGTGCAGCTAAAGTTAGAATGGAATTTGAATCAGATCCCTGATGATACA AGGAAAGAGAAGGAGGTACTTGAGAATCTACAACCTTCCAAACACTTGAAGGATTTGTCA ATCAATAACTATGGTGGTACACAATTTCCAAGTTGGTTATCGGATGTGAATGTGGTGTCC TTACGTTTGGAGGACCGTAAATATTGTGTTGTTTTGCCTCCCTTTGGACTTTTGCCATTT

CTGAAGGAGCTGATGATTAAAGGGCTTGATGGGATAGTGAGTATTGGTGCTGAATTTTAT GGGACTAGCTCTTCTTCATTTACATCCTTGGAAGGATTAGAATTATGCAATATGAAGGAA TGGGAAGAATGGGAATGTAAAGCTGCTTTTCCACGTCTTCAATATCTTTCTTTAGATGAA TACTCAAGCTGA

>AFSP01054890.1_contig55006 FGENESH: 2 1 exon (s) 1402 - 2013 203 aa, chain + Domain - NBS-LRR MPMHLGRLKTLQVLSSFYVGKSCEFNINQLKGLNLHGGLSIGELQNIVNPADAFAASLKN KTHLVQLKLEWNLNQIPDDTRKEKEVLENLQPSKHLKDLSINNYGGTQFPSWLSDVNVVS LRLEDRKYCVVLPPFGLLPFLKELMIKGLDGIVSIGAEFYGTSSSSFTSLEGLELCNMKE WEEWECKAAFPRLQYLSLDEYSS

OLIGO start len tm gc% any 3' seq

LEFT PRIMER 340 20 60.39 50.00 3.00 1.00 TCGGATGTGAATGTGGTGTC

RIGHT PRIMER 556 20 59.96 45.00 2.00 0.00 ATTCCCATTCTTCCCATTCC

SEQUENCE SIZE: 612

PRODUCT SIZE: 217 ####################################################################################

>AFSP01005867.1_contig05872 FGENESH:[mRNA] 1 1 exon (s) 237 - 1988 1752 bp, chain - ATGACGCCGGAAACTCACGTCACTCCTCCTCCACCGTCCTTCAGGCTCCGCTGTGACGTC TTCCTCAGCTTCCGCAGATACGACACGTGCCACACCTTCACCATCAACCTCCAACGAGCG CTCCTCGGCCTCGGCCTCGGCCTCCGCGTCTTCCCTGACGACCATGGCCTCGAGCGCGGC GGCGATATCCAGAAGAACCTTCTGGCGGCCATCGAAGACTCTGCGGCCTGCGTGGTGGTT CTCTCTCCCGATTACGCGTCCTCGCACTGGTGCCTGGAAGAACTGGCCAAGATTTGCGAG GTTGAGAGGCTCGTCCTTCCCGTATTCTACTGCGTCGACCCTTCGGATGTCAGAAAGCAG AAGGGTCGTTCCTTCGAGGGAGCTTTCGCGTCCCATGCAGAGAGGTTTTCCCAAGAGAGT GTTCAGCAGTGGAGGGATGCCATGAAAAAAGTGGGAGGAATCGCTGGTTATGTTGTTGAT GAACAAAGGTTGAACGTTTTGTTTTGTTTTGTTTTTGACAAGCATGTGTCGTGTGATGCT TTTGAACTGTGTCGATTCACTCGTTTTCTGTTTTGTTTTTTCAGTGATAGTGACAAGATT GATAACCTCATTCAGATTTTGGTGCAAACGCTTCTGAAGCAGATGAGAAATACACCTCTG AATGTGGCTCCGTACACAGTTGGGCTAGAAGAAAGAGTCGAAGAACTGAAAAGGCTGTTA

GACGTAAAATCCAACGATGTAAGAGTTCTGGGGTTGTACGGCATGGGTGGGGTTGGTAAA ACAACCCTTGCCAAGACCCTCTTCAACACCCTCGTCGTTCACAATTTCGAGCGTCGCAGT TTCATCCCAAATGTTAGATCACAAGTCTCCAAACACGGTGGTTTGGTTTCTCTCCAAAAC ACACTTCGCAGTGATCTTTCCCAGAGTGGAAAAGACTCAAAAGAGGGTCTCATAAATGAT GTAAACGATGGCATTGCTGCTATCAAAAGAATAGTGCAAGAGAATCGAGTTTTGTTGATC TTAGATGACGTGGATGATGTGGAGCATCTTAACTTTTTGATGGGTAAGAGGGAATGGTTT CACAAAGGAAGCCGAGTTGTGATAACCACAAGGGACAAAGAAGTTTTACGTGAGAGTTAT GTGGACATGCACTATGAGGTGAAGGAGTTGAAATTCTCAGCAGCACTAGAACTATTTTGT TACCATGCAATGAGAAGAAAGGAACCTGCAGAGGGTTTTTTGGATCTTGCAAACCAAATT GTGGAGAAGACTCAAGGGTTGCCCTTGGCCTTGGAAGTTTTTGGTTCTTTTCTGTTTGAC

AAGAGGACGGAGAGGGAATGGAAAGATGCAGTGGAGAAGGTGAAGCAGATTCGACCAGCG TGTCTTCATGATGTGCTGAAGATAAGCTTTGATGCGTTGGATGAAGAAGAGAAGTGTGTG TTCCTTGACATGGCTTGTTTGTTTGTGCAAATGGAAATGAAGAGAGAGGATGTGGTTGAT GTGTTGAATGGGTGTGGTTTTAGTGGGGAGATAGCAGTCACTCTGCTTACAGCAAGGTGT TTGATTAAGATCAGTGGTGATGGTAACGTGTGGATGCATGATCAAGTTAGAGACATGGGG AGGCAGATTGTGGTTAGTGAAAACCTTGCAGATGCTGGTCTACGTAGCAGGCTTTGGGAT

CGTGGTGAAATCTTGACCGTGTTGAAGAGTATGAAGGTAAAGGTTAAATATTATATTGTA GCACAGTTTTGA

>AFSP01005867.1_contig05872 FGENESH: 1 1 exon (s) 237 - 1988 583 aa, chain - Domain - NB-ARC, Toll/Interl ukin receptor TIR domain MTPETHVTPPPPSFRLRCDVFLSFRRYDTCHTFTINLQRALLGLGLGLRVFPDDHGLERG GDIQKNLLAAIEDSAACVVVLSPDYASSHWCLEELAKICEVERLVLPVFYCVDPSDVRKQ KGRSFEGAFASHAERFSQESVQQWRDAMKKVGGIAGYVVDEQRLNVLFCFVFDKHVSCDA FELCRFTRFLFCFFSDSDKIDNLIQILVQTLLKQMRNTPLNVAPYTVGLEERVEELKRLL DVKSNDVRVLGLYGMGGVGKTTLAKTLFNTLVVHNFERRSFIPNVRSQVSKHGGLVSLQN TLRSDLSQSGKDSKEGLINDVNDGIAAIKRIVQENRVLLILDDVDDVEHLNFLMGKREWF HKGSRVVITTRDKEVLRESYVDMHYEVKELKFSAALELFCYHAMRRKEPAEGFLDLANQI VEKTQGLPLALEVFGSFLFDKRTEREWKDAVEKVKQIRPACLHDVLKISFDALDEEEKCV FLDMACLFVQMEMKREDVVDVLNGCGFSGEIAVTLLTARCLIKISGDGNVWMHDQVRDMG

RQIVVSENLADAGLRSRLWDRGEILTVLKSMKVKVKYYIVAQF

OLIGO start len tm gc% any 3' seq

LEFT PRIMER 874 20 60.01 50.00 5.00 0.00 CACGGTGGTTTGGTTTCTCT

RIGHT PRIMER 1098 20 59.85 45.00 3.00 1.00 AACTCGGCTTCCTTTGTGAA

SEQUENCE SIZE: 1752

PRODUCT SIZE: 225 ####################################################################################

>AFSP01018905.1_contig18942 FGENESH:[mRNA] 1 4 exon (s) 1059 - 4217 906 bp, chain + ATGGGATCTGCCAATGAAGAATCCTCTTCTTCAGGATGGTCGAACCATGTTTTCTTGAGT TTTAGGGGTGATGACACACGAAAGGGTTTCACAGACCATCTTTTTGCTTCACTAGAGAGA AGGGGGATCAAAACATTCAAGGATGATCATGATCTCGAGAGGGGAAAAGTGATATCAGTG GAACTCATGAAAGCAATTGAAGAGTCCATGTTTGCACTCATCATTCTCTCACCAAACTAT GCTTCCTCAACATGGTGTTTGGATGAGCTCCAAAAGATTGTAGAGTGTAAGAAAGAAGTT TTTCCAGTCTTCTATGGTGTAGACCCCTCTGATGTGAGGCACCAGAGAGGGAGCTTTGCC GAGGCTTTCAGAGAACATGAAGAGAAATTCAGAGAAGACAGAAAGAAGGTGGGAAAATGG AGAGATGCCTTGAGAGAAGTTGCAGGTTACTCTGGCTGGGACTCCAAGAATCAGCATGAG GCAGCACTGATAGAAACAATAGTTGGACACTTACAACAAAAATTAATTCCTAGATTGCCA TGTTGCACTGATAACCTTGTTGGGATTGATTCACGGATGGAGGAAGTGATTTCACTCATG GGCACAGGTCTAAATGATGTTCGCTTCATAGGAATATGGGGCATGGGGGGAATAGGTAAG

TCAACCATTGCTAGATTAGTATACGAAGCAATCAAAGAGGAATTCAAGGGAACTGATGAA ATTCAAGGCATAGTTCTGAATTTAGTTCAATCGTCTGATTATGAAGCACGCTGGAGCACT GGAGCCTTCTCCAAGATAAGCCAGCTAAGAGTTTGGGGAAAGCATGGAACATTTGTCAAT GCTTTCTTTAGAGGGGACTGCTATAACAAAACTACCCTCATCGTTGGGATGTCTAGTTGG CCTTAA

>AFSP01018905.1_contig18942 FGENESH: 1 4 exon (s) 1059 - 4217 301 aa, chain + Domain - NB-ARC, Toll/Int rleukin receptor homology (TIR) domain MGSANEESSSSGWSNHVFLSFRGDDTRKGFTDHLFASLERRGIKTFKDDHDLERGKVISV ELMKAIEESMFALIILSPNYASSTWCLDELQKIVECKKEVFPVFYGVDPSDVRHQRGSFA EAFREHEEKFREDRKKVGKWRDALREVAGYSGWDSKNQHEAALIETIVGHLQQKLIPRLP

CCTDNLVGIDSRMEEVISLMGTGLNDVRFIGIWGMGGIGKSTIARLVYEAIKEEFKGTDE IQGIVLNLVQSSDYEARWSTGAFSKISQLRVWGKHGTFVNAFFRGDCYNKTTLIVGMSSW P

OLIGO start len tm gc% any 3' seq

LEFT PRIMER 153 20 59.92 50.00 8.00 1.00 TCTCGAGAGGGGAAAAGTGA

RIGHT PRIMER 349 20 59.98 55.00 3.00 1.00 CTCTCTGGTGCCTCACATCA

SEQUENCE SIZE: 906

PRODUCT SIZE: 197 ####################################################################################

>AFSP01069439.1_contig69556 FGENESH:[mRNA] 1 3 exon (s) 31 - 2442 1689 bp, chain - TCTCCGTGGTGCTTGAATGAGTTAGGGAAAATAATGGAGTGCCACAGAACCATAGGGCAC AAAGTTCTCCCGGTGTTCTATGATGTTTATCCCTCCGAAGTGCGCCATCAAAAAGGCGAG TTTGGAAAAGCACTCGAAAAACTTCAGGAGAGGATTTTAAGCTACGTGAATGAGGAGTTT CCGCAATTGGTGCGTGGGATTGGTGACAAATCGGAAGTGATGGATTCCCGATTGAAGGTC ATGTTCCCGAATTGTAGGAAGGCACTTCGTGAGGCCGCTGGCATGTCAGGTGTTGTAGTA CTCAACTCCAGAAATGAAAGGGAGACTATAAAAAATATTGTTGAAAATGTTGCACATTTG TTAGACAAGACAGAGTTATTTGTTGCCAATAATCCAGTGGGAGTAGAACCTCGAGTACAA GCAATGAGTCAATTGCTACTCCAAAAATCATCAAATGATGTCCTACTATTAGGGATGTGG GGGATGGGAGGCATTGGTAAAACAACCATTGCTAAAGCCATTTACAATAAGATTGGCCGC AATTTTGAGGGAAGGAGCTTCCTTGCAAATATCAGGGAAGTTTGGAGGCAAGACGTTGGC CAAGTCAATTTACAAGAACAACTTCTGTCTGATATCAACAAAGAAACAAAAATAACGATA CATAATGCTGAATCAGGAAAAAATATATTAAAAAAAATACTTTGCTATAAAAAGGTACTT CTTGTATTGGATGATGTGAATGAATTGGACCAACTAAATGCTTTATGTGGAAGTTACAAA TGGTTTGGTCCAGGTAGTAGAATAATCATTACCACTAGAGATCAAGATATACTCAGAGGG AGAGTTGACATAGTATATACAATGAAAGGCATGGATGAAGGTGAATCCATTGAGCTTTTT AGTTGGCACGCATTCAAGCAAGCAAGTCCTAAAGAAAATTTTACCCAACTTTCTAGAAAT GTAATTACGTATTCCGGGGGATTGCCACTAGCCCTTGAAGTGCTTGGGTGTTATTTGTTT GATATGAAGGTAGCAGAGTGGGAGAATGTATTGGAGAAACTTAAGAGAATTCCTAATCAT CAAGTACAAAAGAAGTTAAAAATAAGCTATGATGGTTTAAATGATGATACTGAGAGAGAT ATATTCCTCGATATAGCTTGTTTCTTTATTGGCATGGATCGGAATGATGTTATACATATA TTAAATGGTTGTGGGCTTTTCGCAGAAAATGGAATACGTGTCCTTGTAGAGCGAAGCCTT GTAACAATTGATAATAAAAACAAGCTTCGAATGCATGATTTGATAAGGGACATGGGAAGG GAAATCGTTCGTGCAAATTCACTAAAGGTTCTTGAGAAGCGTAGCAGGTTATGGTTTCAT GAGGATGCGCTTGATGTATTAGCAAAAGAAACGGGAACAGAAGCTATTGAGGGACTGGCT TTGAGGTTATCAATAAATAATGCAACATGTTTGAGCACTAAAGCTTTTAAAAACATGGAG AAACTTAGGTTGCTTCAACTTGCTGGTGTAAGACTTGATGGAGATTTTAAATATCTTTCC AAAGATCTTAGATGGCTGTGTTGGCATGGATTTCCTTTAAAATACATACCAACAAACTTT TATCAAAGAAATCTTATTTCAATTGAGTTACAAAACAGTAATGTTCAACTTGTGTGGAAA

GAAGCTCAG

>AFSP01069439.1_contig69556 FGENESH: 1 3 exon (s) 31 - 2442 563 aa, chain - Domain - NB-ARC, Toll/Interle kin receptor homology (TIR) domain SPWCLNELGKIMECHRTIGHKVLPVFYDVYPSEVRHQKGEFGKALEKLQERILSYVNEEF PQLVRGIGDKSEVMDSRLKVMFPNCRKALREAAGMSGVVVLNSRNERETIKNIVENVAHL LDKTELFVANNPVGVEPRVQAMSQLLLQKSSNDVLLLGMWGMGGIGKTTIAKAIYNKIGR NFEGRSFLANIREVWRQDVGQVNLQEQLLSDINKETKITIHNAESGKNILKKILCYKKVL LVLDDVNELDQLNALCGSYKWFGPGSRIIITTRDQDILRGRVDIVYTMKGMDEGESIELF SWHAFKQASPKENFTQLSRNVITYSGGLPLALEVLGCYLFDMKVAEWENVLEKLKRIPNH QVQKKLKISYDGLNDDTERDIFLDIACFFIGMDRNDVIHILNGCGLFAENGIRVLVERSL VTIDNKNKLRMHDLIRDMGREIVRANSLKVLEKRSRLWFHEDALDVLAKETGTEAIEGLA LRLSINNATCLSTKAFKNMEKLRLLQLAGVRLDGDFKYLSKDLRWLCWHGFPLKYIPTNF YQRNLISIELQNSNVQLVWKEAQ

OLIGO start len tm gc% any 3' seq

LEFT PRIMER 58 20 60.01 50.00 4.00 0.00 CACAAAGTTCTCCCGGTGTT

RIGHT PRIMER 220 20 60.09 45.00 3.00 3.00 TCACTTCCGATTTGTCACCA

SEQUENCE SIZE: 1689 INCLUDED REGION SIZE: 1689 PRODUCT SIZE: 163

####################################################################################

>gi|7107255|gb|AF186634.1| Cajanus cajan clone PP1 unknown gene 516 bp GGTGGGGTTGGGAAGACAACGCTTGCCCAACATGTATACAATGATCCAAGGATGGAGGAGGCTAACT TG GCATCAAAGCTTGGGTTTGTGCTTCGAATGATTTTGATGTTTTGACAGTATCGAAAAAAATTCTTGAGG

AATCACTAAATCAAAAGATGATAGTGGAGACCTAGAAATGGTTCATGGAAGATTGAAAGAAAAGTTG CA GGGAAGATATTTCTTTTGGTTTTAGATGATGTTTGGAATGAAAGACGGGAGAAATGGGAAGTTGTGCA A

CTCCTCTCATTTATGGGGCACAAGGAAGTAAAATTCTTGTCACGACACGAAGTAAGAAAGTTTCTTCG T GATGCAGTCAAATAAAGTACATCACCTAAAGCAATTACAAGAAGATCATAGTTGGCAAGTTTTCGCCA A

CACGCACTCCAAGATGATAATACTCAAGCAAATTTTGAATTGAAGGAGATTGGTATAAAAATAGTTAA A

AGTGTAAAGGATTGCCACTAGCGTTG

>gi|7107256|gb|AAF36342.1|AF186634_1 unknown, partial [Cajanus cajan] 172 aa Domain - NB-ARC GGVGKTTLAQHVYNDPRMEEANFGIKAWVCASNDFDVLTVSKKILEAITKSKDDSGDLEMVHGRLKEKL

GKIFLLVLDDVWNERREKWEVVQTPLIYGAQGSKILVTTRSKKVSSMMQSNKVHHLKQLQEDHSWQVFA HALQDDNTQANFELKEIGIKIVKKCKGLPLAL

OLIGO start len tm gc% any 3' seq

LEFT PRIMER 83 20 59.94 45.00 6.00 3.00 GGGTTTGTGCTTCGAATGAT

RIGHT PRIMER 300 20 60.03 50.00 4.00 0.00 TGCCCCATAAATGAGAGGAG

SEQUENCE SIZE: 516

PRODUCT SIZE: 218 ######################################################################################

>gi|7107257|gb|AF186635.1| Cajanus cajan clone PP2 unknown gene 492 bp GGTGTTGGTAAGACAACTCTAGCAAAGGCAATTTATTATCACAAAGCAGCCGTTGAGCACTTCCCGAT C GTGTCTGGGTGACAGTAACTGAAGGAGCTGCCTACAAAGCACAAGTTCTGCTGATGAAAAAGGATGG AC TAAAGACCAGACATTGTATGTCACTCAGGTACGTGATCACTTGAAAGAGAAGCTGTGCCTTGTTGTTCT

GATAATGTATCAAACACAAAAGATTTTGATAAACTGTATGAAATATTATCTGGATCTGGAATGATAAA G GGAGCAGAGTAGTGCTGACGACACGCTTTAAGAATGTAGCTTTACATGCTGACACAAGTAACACCCCT A

CCAAATTCGACTGCTAACAAAGGAAGAGAGTTGGGAGTTGTTTAAGAAGGTGACAGGCACCGAGAAG CC AAATTAGAATCAAAAGTGGAAAAACTTGCAAGAAATGTCGTGGGAAGATGCGGGGGGCTACCACTAG AC

TG

>gi|7107258|gb|AAF36343.1|AF186635_1 unknown, partial [Cajanus cajan] 164 aa Domain - NB-ARC GVGKTTLAKAIYYHKAAVEHFPIRVWVTVTEGAAYKAQVLLMKKDGTKDQTLYVTQVRDHLKEKLCLV L DNVSNTKDFDKLYEILSGSGMINGSRVVLTTRFKNVALHADTSNTPHQIRLLTKEESWELFKKVTGTEKT KLESKVEKLARNVVGRCGGLPLAL

OLIGO start len tm gc% any 3' seq

LEFT PRIMER 68 20 60.15 55.00 4.00 2.00 TCCGTGTCTGGGTGACAGTA

RIGHT PRIMER 311 20 60.06 50.00 4.00 3.00 TTAAAGCGTGTCGTCAGCAC

SEQUENCE SIZE: 492

PRODUCT SIZE: 244 ######################################################################################

>gi|7107259|gb|AF186636.1| Cajanus cajan clone PP3 unknown gene 510 bp GGGGGGGTGGGGAAGACGACTCTTGCTACTGTTTTGTATCATAGAATTTGTCATCAATATACTGCTCGT

GTTTCATCGACGACTTAAGCAAAGTTTATAGAGACTTTGGTCCAATTGGTGCACTAAAGCAATTGCTTT

TCAAACTCTAAATGAAGAGAACCTTCATATAAGCAATCTTTATAACGCAGCCAATTTGATACGAAGTA G

CTACGTTATGTAAAGACCCTTATAGTTCTCGATAATGTTGATGAAGTTGAACAACTAGAGAAGTTAGTT

TGGATCGCCAATGGTTTGGTGCAGGGAGTAGAATCATCATAATTTCTAGAAACAAGCGTATCTTAGAA A GTATGGAGTGGATGTAGTCTACAAAGTTCAACTCTTAAATAGTACTAATGCTCTTAAATTGTTTTGTAA

AAGGCTTTTCATAGTGAAGATATTGTGTATGATTACAAAAGTTTGACAAATGATGTGCTACAATATGCT

AAGGCCTCCCCCTCGCCCTC

>gi|7107260|gb|AAF36344.1|AF186636_1 unknown, partial [Cajanus cajan] 170 aa Domain - NB-ARC GGVGKTTLATVLYHRICHQYTARCFIDDLSKVYRDFGPIGALKQLLCQTLNEENLHISNLYNAANLIRSR LRYVKTLIVLDNVDEVEQLEKLVVDRQWFGAGSRIIIISRNKRILEKYGVDVVYKVQLLNSTNALKLFCK KAFHSEDIVYDYKSLTNDVLQYAKGLPLAL

OLIGO start len tm gc% any 3' seq

LEFT PRIMER 293 20 60.11 55.00 4.00 0.00 GGTTTGGTGCAGGGAGTAGA

RIGHT PRIMER 501 20 59.42 50.00 6.00 2.00 GGGGAGGCCTTTAGCATATT

SEQUENCE SIZE: 510

PRODUCT SIZE: 209 ######################################################################################
