# Supplementary material for: Refinement of Draft Genome Assemblies of Pigeonpea (Cajanus cajan)
Source: Front Genet. 2020 Dec 15;11:607432. doi: 10.3389/fgene.2020.607432 (PMC7770131; doi:10.3389/fgene.2020.607432)
Supplement: Supplementary Table 2 — Putative disease resistance genes predicted from the improved reassembly of pigeonpea. [file Table_2.DOCX]

**Supplementary Table 1:** BUSCO (Benchmarking Universal Single-Copy Orthologs) genes distribution.

| **BUSCOs orthologs** | **A1 Assembly** | **A2 Assembly** | **A3 Assembly** |
| --- | --- | --- | --- |
| **Single copy** | 1107 | 1165 | 1321 |
| **Duplicated** | 121 | 101 | 33 |
| **Fragmented** | 81 | 72 | 57 |
| **Missing** | 131 | 102 | 29 |
