# Supplementary material for: Refinement of Draft Genome Assemblies of Pigeonpea (Cajanus cajan)
Source: Front Genet. 2020 Dec 15;11:607432. doi: 10.3389/fgene.2020.607432 (PMC7770131; doi:10.3389/fgene.2020.607432)
Supplement: Supplementary Table 3 — Numbers of predominant SSRs. [file Table_3.DOCX]

**Supplementary Table 2: Putative Disease resistance genes predicted from Improved reassembly of Pigeonpea**

| Contig | Protein | Length | TargetP | HMMTOP | SignalP | Domain (Interproscan) |
| --- | --- | --- | --- | --- | --- | --- |
| contig01377  AFSP01001376.1 | Contig01377.1 | 141 aa | SP | 3 helices  (70-87 96-113 124-141) | No | signal peptide, transmembrane regions |
|  | Contig01377.2  (393 bp) | 130 aa | other | 0 | No | NB-ARC |
|  | Contig01377.3 | 35 aa | other | 0 | No | No hits |
| contig02015  AFSP01002013.1 | Contig02015.1 | 218 aa | CP | 0 | No | Glucosyltransferases, membrane-associated proteins |
|  | Contig02015.2 | 104 aa | other | 1 (18-40 ) | No | Trnasmembrane region |
|  | Contig02015.3  (672 bp) | 223 aa | other | 1 (104-122 ) | No | NB-ARC, NBS_LRR |
|  | Contig02015.4 | 191 aa | other | 0 | No | No hits |
| contig05554  AFSP01005549.1 | Contig05554.1 | 182 aa | other | 0 | No | Ribosomal protein L5 |
|  | Contig05554.2 | 37 aa | MP | 0 | No | No hits |
|  | Contig05554.3 | 1273 aa | SP | 0 | No | NBS-LRR, NB_ARC |
| contig05714  AFSP01005709.1 | Contig05714.1 | 45 aa | SP | 1 (4-20) | No | signal peptide, transmembrane region |
|  | Contig05714.2 | 271 aa | other | 0 | No | DNA/RNA polymerase |
|  | Contig05714.3 | 106 aa | CP | 0 | No | No hits |
|  | Contig05714.4 | 313 aa | other | 0 | No | Ribonuclease H-like domain, nucleic acid binding |
|  | Contig05714.5 | 32 aa | other | 0 | No | No hits |
|  | Contig05714.6 | 1117 aa | SP | 0 | No | NB-ARC, NBS LRR |
| contig06314  AFSP01006309.1 | Contig06314.1 | 1221 aa | SP | 1 (194-211 ) | No | NB-ARC, NBS-LRR |
|  | Contig06314.2 | 115 aa | other | 0 | No | No hits |
|  | Contig06314.3 | 85 aa | SP | 0 | No | No hits |
|  | Contig06314.4 | 174 aa | other | 0 | No | No hits |
| contig15070  AFSP01015044.1 | Contig15070.1 | 325 aa | other | 2 (67-84 205-222) | No | Reverse transcriptase, DNA polymerase |
|  | Contig15070.2 | 1227 aa | SP | 0 | No | NBS_LRR, NB-ARC |
| contig23458  AFSP01023410.1 | contig23458 | 1195 aa | other | 0 | No | NB_ARC, NBS_LRR |
|  | contig23458 | 223 aa | other | 0 | No | Peptidase A2A |
|  | contig23458 | 132 aa | other | 0 | No | No hits |
| contig29789  AFSP01029727.1 | Contig29789.1  (2040 bp) | 679 aa | SP | 0 | Yes | NB_ARC, NBS-LRR, signal peptide |
| contig32340  AFSP01032261.1 | Contig32340.1 | 795 aa | other | 0 | No | NB_ARC, LRR |
| contig39330  AFSP01039224.1 | Contig39330.1 | 1280 aa | SP | 0 | No | NB-ARC, NBS-LRR |
| contig39609  AFSP01039503.1 | Contig39609.1 | 1213 aa | other | 0 | No | NB-ARC, NBS-LRR |
| contig42860  AFSP01042748.1 | Contig42860.1 | 1207 aa | SP | 0 | No | NB-ARC, NBS-LRR |
| contig43480  AFSP01043367.1 | Contig43480.1 | 1195 aa | other | 0 | No | NB_ARC, NBS_LRR |
| contig46052  AFSP01045937.1 | Contig46052.1 | 211 aa | other | 0 | No | Zinc finger like domain |
| contig53527  AFSP01053411.1 | Contig53527.1 | 774 aa | other | 0 | No | NB_ARC |
| contig55006  AFSP01054890.1 | Contig55006.1 | 115 aa | other | 0 | No | No hits |
|  | Contig55006.2  (612 bp) | 203 aa | SP | 0 | No | NBS-LRR |
|  | Contig55006.3 | 291 aa | other | 0 | No | LRR domain |
| contig89364  AFSP01089246.1 | Contig89364.1 | 430 aa | SP | 0 | No | signal peptide |
| contig03389  AFSP01003385.1 | Contig03389.1 | 241 aa | CP | 2(191-207 216-232) | No | transmembrane region |
|  | Contig03389.2 | 40 aa | other | 0 | No | No hits |
|  | Contig03389.3 | 941 aa | other | 0 | No | NBS-LRR, NB-ARC, Toll/interleukin-1 receptor homology (TIR) domain, ATPase |
| contig05338  AFSP01005333.1 | Contig05338.1 | 905 aa | other | 0 | No | NB-ARC, LRR |
|  | Contig05338.2 | 124 aa | other | 0 | No | No hits |
| contig05872  AFSP01005867.1 | Contig05872.1  (1752 bp) | 583 aa | MP | 0 | No | NB-ARC, Toll/Interleukin receptor TIR domain |
| contig07729  AFSP01007721.1 | Contig07729.1 | 766 aa | MP | 0 | No | NB-ARC, LRR |
| contig09910  AFSP01009896.1 | Contig09910.1 | 1061 aa | SP | 0 | No | NB-ARC, LRR, Toll/Interleukin receptor TIR domain |
|  | Contig09910.2 | 145 aa | SP | 1 (6-26) | Yes | signal peptide, transmembrane region |
| contig14273  AFSP01014249.1 | Contig14273.1 | 203 aa | other | 0 | No | No hits |
|  | Contig14273.2 | 1025 aa | other | 0 | No | NB-ARC, NBS-LRR, Toll/Interleukin receptor TIR domain |
| contig18942  AFSP01018905.1 | Contig18942.1  (906 bp) | 301 aa | other | 0 | No | NB-ARC, Toll/Interleukin receptor homology (TIR) domain |
|  | Contig18942.2 | 359 aa | CP | 2(75-95 112-133 ) | No | Ferric reductase, NAD binding, Flavoprotein transmembrane component |
|  | Contig18942.3 | 524 aa | CP | 1 (154-172 ) | No | No hits found |
|  | Contig18942.4 | 1132 aa | MP | 0 | No | Reverse transcriptase, Ribonuclease H domain, Integrase, catalytic core |
|  | Contig18942.5 | 35 aa | other | 0 | No | No hits |
| contig21577  AFSP01021533.1 | Contig21577.1 | 98 aa | other | 0 | No | Nicotinamide N-methyltransferase |
|  | Contig21577.2 | 255 aa | SP | 0 | No | Retrovirus zinc finger-like domains, Zinc finger, CCHC-type |
|  | Contig21577.3 | 272 aa | other | 1 (120-137 ) | No | Nicotinamide N-methyltransferase, putative, S-adenosyl-L-methionine-dependent methyltransferases |
| contig21911  AFSP01021867.1 | contig21911 | 85 aa | other | 0 | No | No hits |
|  | contig21911 | 924 aa | other | 0 | No | Ribonuclease H-like, Retrovirus zinc finger-like domains, Reverse transcriptase, RNA-dependent DNA polymerase |
|  | contig21911 | 229 aa | MP | 1 (34-52 ) | No | signal peptide |
|  | contig21911 | 881 aa | other | 0 | No | NB-ARC, NBS-LRR, ATPase, Toll/Interleukin receptor homology (TIR) domain |
| contig23510  AFSP01023462.1 | contig23510 | 278 aa | other | 0 | No | Arabidopsis retrotransposon Orf1 |
| contig26980  AFSP01026927.1 | Contig26980.1 | 1260 aa | SP | 0 | No | NB-ARC, NBS-LRR, Signal peptide |
| contig35942  AFSP01035846.1 | Contig35942.1 | 663 aa | other | 0 | No | NB-ARC, Toll/Interleukin receptor homology (TIR) domain |
| contig39988  AFSP01039880.1 | Contig39988.1 | 1070 aa | other | 0 | No | NB-ARC, NBS-LRR, Toll/Interleukin receptor homology (TIR) domain |
| contig40008  AFSP01039900.1 | Contig40008.1 | 809 aa | MP | 0 | No | NB-ARC, signal peptide, Toll/Interleukin receptor homology (TIR) domain |
| contig40995  AFSP01040886.1 | Contig40995.1 | 1159 aa | other | 1(195-212) | No | NB-ARC, NBS-LRR |
| contig4333  AFSP01043219.1 | Contig4333.1 | 663 aa | SP | 1(10-27 ) | Yes | Serine-threonine/tyrosine-protein kinase catalytic domain, signal peptide, transmembrane region |
| contig48397  AFSP01048281.1 | Contig48397.1 | 902 aa | other | 0 | No | NB-ARC, Toll/Interleukin receptor homology (TIR) domain |
| contig61883  AFSP01061766.1 | Contig61883.1 | 81 aa | other | 0 | No | No hits |
| contig69556  AFSP01069439.1 | Contig69556  (1689 bp) | 563 aa | other | 0 | No | NB-ARC, Toll/Interleukin receptor homology (TIR) domain |
| Contig06142  .1  AFSP01006137.1 | Contig06142.1 | 272 aa | SP | 1(6-25) | Yes | Glycoside hydrolase, Chitin-binding, type, Lysozyme-like domain |
|  | Contig06142.2 | 358 aa | MP | 2(311-330 337-356) | No | transmembrane region |
|  | Contig06142.3 | 480 aa | other | 1(16-33 ) | No | Pentatricopeptide repeat, Tetratricopeptide-like helical |
| Contig14710  .1  AFSP01014686.1 | Contig14710.1 | 182 aa | other | 0 | No | Ubiquitin |
|  | Contig14710.2 | 291 aa | other | 0 | No | No hits |
| Contig15106  .3  AFSP01015080.1 | Contig15106.1 | 714 aa | other | 0 | No | Zinc finger, Reverse transcriptase, RNA-dependent DNA polymerase |
|  | Contig15106.2 | 825 aa | MP | 0 | No | Pentatricopeptide repeat |
|  | Contig15106.3 | 214 aa | other | 1(170-187 ) | No | hemimethylated DNA binding domain 83 |
|  | Contig15106.4 | 147 aa | other | 0 | No | Ribosomal protein S14 |
|  | Contig15106.5 | 133 aa | CP | 0 | No | No hits |
|  | Contig15106.6 | 327 aa | other | 0 | No | Glycoside hydrolase, chitinase active site |
| contig36637  AFSP01036539 | Contig36637.1 | 132 aa | MP | 0 | No | No hits |
|  | Contig36637.2 | 838 aa | SP | 2(376-394 403-422 ) | Yes | Serine-threonine/tyrosine-protein kinase, Glycoside hydrolase |
| contig38046  AFSP01037943.1 | Contig38046.1 | 37 aa | other | 0 | No | Signal peptide |
|  | Contig38046.2 | 326 aa | SP | 1(7-24 ) | Yes | chitin binding |
|  | Contig38046.3 | 24 aa | other | 0 | No | No hits |
| contig42269  AFSP01042158.1 | Contig42269.1 | 331 aa | other | 0 | No | Membrane attack complex component/perforin/complement C9 |
| contig134574  AFSP01134431 | Contig134574.1 | 134 aa | other | 0 | No | Lysozyme-like domain, Glycoside hydrolase, family 19, catalytic |
| contig12564  AFSP01012543.1 | Contig12564.1 | 92 aa | other | 0 | No | Peptide methionine sulphoxide reductase MsrA |
|  | Contig12564.1 | 362 aa | SP | 1(6-23) | Yes | Signal peptide, transmembrane regions, Proteinase inhibitor I29, cathepsin propeptide |
| contig18626  AFSP01018589.1 | Contig18626.1 | 197 aa | other | 1(176-195 ) | No | Peptidase aspartic |
| contig18627  AFSP01018590.1 | Contig18627.1 | 275 aa | SP | 1(7-29) | Yes | Peptidase aspartic |
| contig33506  AFSP01033418.1 | Contig33506.1 | 275 aa | SP | 1(7-29) | Yes | Peptidase aspartic |
| contig40713  AFSP01040604.1 | Contig40713.1 | 461 aa | SP | 2( 7-29 50-69) | No | Signal peptide, transmembrane region, Peptidase aspartic |
| contig01485  AFSP01001484.1 | Contig01485.1 | 289 aa | MP | 0 | No | signal peptide |
|  | Contig01485.2 | 617 aa | MP | 0 | No | Pentatricopeptide repeat, Tetratricopeptide-like helical |
|  | Contig01485.3 | 146 aa | other | 0 | No | Glycosyl hydrolase domain |
| contig04803  AFSP01004798.1 | Contig04803.1 | 84 aa | CP | 0 | No | no hits |
|  | Contig04803.2 | 716 aa | MP | 1(20-39) |  | Armadillo/beta-catenin-like repeats, ubiquitin ligase complex |
| Contig31936  AFSP01031858.1 | Contig31936.1 | 776 aa | other | 329-48 305-322 331-349 ) |  | Aldolase-type TIM barrel, Raffinose synthase |
| Contig37308  AFSP01037207.1 | Contig37308.1 | 714 aa | MP | 1 (20-39 ) |  | Armadillo, ubiquitin-protein ligase activity, Zinc finger |
| contig93833  AFSP01093713.1 | Contig93833.1 | 157 aa | other | 0 |  | Glycoside hydrolase, Aldolase-type TIM barrel |
| contig35533  AFSP01035438.1 | Contig35533.1 | 312 aa | other | 0 |  | Glycoside hydrolase |
|  | Contig35533.2 | 55 aa | other | 0 |  | No hits |
