# Supplementary material for: Refinement of Draft Genome Assemblies of Pigeonpea (Cajanus cajan)
Source: Front Genet. 2020 Dec 15;11:607432. doi: 10.3389/fgene.2020.607432 (PMC7770131; doi:10.3389/fgene.2020.607432)
Supplement: Supplementary Table 7 — List of primer sequences used in PCR amplification. [file Table_7.DOCX]

**Supplementary Table 7: List of Pigeon pea disease resistance genes submitted to NCBI**

| **Accession No.** | **Domain information** |
| --- | --- |
| KF130783.1 | NBS-LRR disease resistance domain |
| KF130784.1 | NBS-LRR disease resistance domain |
| KF130785.1 | NBS-LRR disease resistance domain |
| KF130786.1 | NBS-LRR disease resistance domain |
| KC966926.1 | NBS-LRR disease resistance domain |
| KC966925.1 | NBS-LRR disease resistance domain |
| KC966923.1 | NBS-LRR disease resistance domain |
| KC966922.1 | NBS-LRR disease resistance domain |
| KC966921.1 | NBS-LRR disease resistance domain |
