# Supplementary material for: Refinement of Draft Genome Assemblies of Pigeonpea (Cajanus cajan)
Source: Front Genet. 2020 Dec 15;11:607432. doi: 10.3389/fgene.2020.607432 (PMC7770131; doi:10.3389/fgene.2020.607432)
Supplement: Supplementary Table 8 — Pigeonpea phenotypic field scores for Fusarium wilt disease reaction. [file Table_8.DOCX]

**Supplementary Table 8: Pigeon pea phenotypic field scores for *Fusarium* wilt disease reaction.**

Mean of data collected during rainy seasons, 2011 and 2012, NBPGR Regional farm, Ranchi, India farm

Based on disease score 0-9 scale.

**Ranchi Farm (2011 & 12) Rahuri Farm ( 2013 & 14)**

| **S.N.** | **Accession** | **Disease score/pressure** | **Field disease reaction** | **Disease score/pressure** | **Field**  **disease reaction** |
| --- | --- | --- | --- | --- | --- |
| **1** | IC73327 | 01 | R | 01 | R |
| **2** | IC74013 | 02 | R | 01 | R |
| **3** | IC73746 | 02 | R | - | Not reported |
| **4** | IC73745 | 02 | R | - | Not reported |
| **5** | IC73329 | 01 | R | 01 | R |
| **6** | IC73341 | 01 | R | 01 | R |
| **7** | IC74049 | 02 | R | 01 | R |
| **8** | IC94036 | 02 | R | - | Not reported |
| **9** | IC73953 | 01 | R | - | Not reported |
| **10** | ICP9174 | 02 | R | - | Not reported |
| **11** | IC73960 | 01 | R | - | Not reported |
| **12** | IC245134 | 02 | R | 01 | R |
| **13** | IC73969 | 02 | R | 02 | R |
| **14** | IC245141 | 02 | R | 02 | R |
| **15** | ICP-5097 | 03 | T | 03 | T |
| **16** | ICP-8865 | 03 | T | - | Not reported |
| **17** | IC245140 | 03 | T | 03 | T |
| **18** | IC245135 | 03 | T | - | Not reported |
| **19** | IPA-16F | 03 | T | - | Not reported |
| **20** | IC245137 | 03 | T | - | Not reported |
| **21** | IC245148 | 05 | S | 05 | S |
| **22** | IC245130 | 05 | S | 05 | S |
| **23** | IC245132 | 05 | S | 05 | S |
| **24** | PT-0307 | 07 | S | 05 | S |
| **25** | PT-03-142 | 07 | S | 05 | S |
| **26** | Asha  ( Check/control) | 01 | R | 01 | R |

Based on field disease score of 0-9, where 1 & 2 – resistant (R), 3 & 4 – tolerant (T) and 5 to 9- disease susceptible (S).

Plot size: row to row-75cm, Plant to plant space-35 cm.
